# Left atrial function is a strong predictor of exercise intolerance in patients with hypertrophic cardiomyopathy

**DOI:** 10.3389/fcvm.2026.1717245

**Published:** 2026-02-03

**Authors:** Mengdi Yu, Lutong Pu, Yaxin Zhou, Yuanwei Xu, Ke Wan, Jiajun Guo, Yangjie Li, Yuchi Han, Jie Wang, Yucheng Chen

**Affiliations:** 1 Department of Cardiology, West China Hospital, Sichuan University, Chengdu, Sichuan, China; 2 Rehabilitation, Medicine Center, West China Hospital, Sichuan University, Chengdu, Sichuan, China; 3 Cardiac Imaging and Target Therapy Lab, West China Hospital, Sichuan University, Chengdu, Sichuan, China; 4 Department of Geriatrics, West China Hospital, Sichuan University, Chengdu, Sichuan, China; 5 Cardiovascular Division, Wexner Medical Center, The Ohio State University, Columbus, OH, United States

**Keywords:** cardiac magnetic resonance, cardiopulmonary exercise test, exercise tolerance, hypertrophic cardiomyopathy, left atrial

## Abstract

**Background:**

Patients with hypertrophic cardiomyopathy (HCM) often experience exercise intolerance. However, the primary determinants of exercise capacity in patients with HCM remain unclear.

**Objectives:**

This study aimed to evaluate exercise intolerance in patients with HCM and investigate the associations between exercise capacity and clinical/imaging characteristics, specially focusing on left atrial (LA) function.

**Methods:**

This retrospective analysis utilized data from a prospective cohort of adult patients with HCM. Included patients underwent standardized cardiopulmonary exercise testing (CPET) and 3.0T cardiac magnetic resonance (CMR) between May 2012 and May 2025. Exercise capacity was primarily assessed by peak oxygen uptake (Peak VO_2_) measured during CPET. LA function (diameter, volumes and strain) was evaluated using CMR. Univariable and multivariable linear regression analyses were used to identify determinants of exercise capacity.

**Results:**

Among 120 participants [60.8% (73/120) male, mean age: 45.5 ± 14.4 years], 77.8% (91/120) had reduced exercise tolerance (Peak VO_2_ < 80% of the predicted normal value). The mean value of Peak VO_2_ was 20.6 ± 5.6 mL/min/kg. Lower Peak VO_2_ correlated with older age, female gender, larger LA size and reduced LA strain. Notably, LV ejection fraction, volume, strain, and outflow tract pressure gradient showed no significant associations (all *P* value >0.05). Besides, multivariate analysis identified LA reservoir strain as an independent predictor of Peak VO_2_ (*β* = 0.275, *P* < 0.001), outperforming LA diameter and volumes.

**Conclusion:**

LA dysfunction, as reflected by reduced strain, independently predicts impaired exercise capacity in HCM.

## 1 Introduction

Hypertrophic cardiomyopathy (HCM), characterized by left ventricular (LV) hypertrophy, is the most common inherited cardiomyopathy, with prevalence of 1:200–1:500. Its manifestations are highly variable, ranging from asymptomatic states to severe functional impairment and even sudden cardiac death (1–3). Exercise intolerance, a hallmark of disease progression, correlates with adverse outcomes (4–6). Cardiopulmonary exercise testing (CPET) is the gold standard for assessing exercise capacity (2, 7). Reduced peak oxygen uptake (Peak VO₂) assessed by CPET is commonly observed in patients with HCM (8). However, the determinants of exercise capacity in patients with HCM are incompletely understood. Furthermore, in the era of cardiac myosin inhibitors treatment, it is necessary to develop new imaging markers to monitor treatment effects and side effects.

The underlying pathophysiological mechanisms of exercise intolerance in HCM patients are complex, involving LV outflow tract (LVOT) obstruction, diastolic dysfunction, systolic dysfunction, chronotropic incompetence, and microvascular dysfunction (8). These factors collectively contribute to elevated LV filling pressures, chronic left atrial (LA) overload, and subsequent LA remodeling (9). Cardiac magnetic resonance (CMR) enables precise evaluation of LA strain, a sensitive marker of myocardial deformation. Although prior studies identified LV-related predictors of exercise tolerance in HCM (10, 11), the role of LA function remains not well understood. Given the crucial role of LA in compensating for increased LV filling pressures, assessment of its function may provide valuable insights into the determinants of exercise capacity in HCM. Therefore, this study investigated associations between CPET parameters, clinical characteristics, and CMR-derived LA/LV metrics to identify key determinants of exercise capacity in HCM.

## 2 Methods

### 2.1 Study population

This retrospective study utilized data from a prospective cohort of adult patients with HCM. Included patients underwent CPET and 3.0T CMR between May 1, 2012, and May 1, 2025. Both CPET and CMR were conducted following standardized protocols during the patients' initial evaluation.

According to the guidelines of the European Society of Cardiology (ESC) (1) and American Heart Association (AHA)/American College of Cardiology (ACC) (2), the diagnosis of HCM in an adult was defined as patients with LV wall thickness (LVWT) ≥15 mm in the absence of other causes of hypertrophy, or LVWT ≥13 mm when present in family members of a patient with HCM or in conjunction with a positive genetic test (2). Hypertrophic obstructive cardiomyopathy (HOCM) was defined as an LV outflow tract (LVOT) pressure gradient (PG) ≥30 mmHg at rest or ≥30 mmHg with provocation or exercise. The exclusion criteria comprised HCM phenocopies (e.g., amyloidosis, Fabry disease, Danon disease), secondary myocardial hypertrophy (e.g., hypertension-related), comorbidities affecting exercise capacity (e.g., chronic obstructive pulmonary disease, moderate to severe anemia and severe coronary artery disease), contraindications for CMR and poor CMR imaging quality due to arrhythmia or motion artifacts. HCM phenocopies were excluded on the basis of clinical presentation, imaging characteristics, laboratory findings, and when clinically indicated, targeted genetic testing or disease-specific confirmatory examinations. The investigation was approved by the ethics committee of West China Hospital, and informed consent was obtained from all participants.

### 2.2 Cardiopulmonary exercise testing

CPET was performed using a symptom-limited incremental exercise test on a cycle ergometer. Workload increments were calculated based on predicted VO₂ and adjusted according to the individual clinical condition, activity level, and cardiopulmonary status (12, 13). Peak VO₂ was determined as the highest 30-second average of oxygen consumption recorded during exercise and was expressed either as an absolute value or as a percentage of the predicted value based on age, sex, and body weight. The anaerobic threshold (AT) is defined as the point during incremental exercise at which anaerobic metabolism increases disproportionately relative to aerobic metabolism, identified by a sharp rise in the slope on the VCO₂/VO₂ graph. Metabolic equivalent (MET) is a measure of exercise intensity, with 1 MET equal to an oxygen consumption of 3.5 mL/kg/min at rest. The higher the VE/VCO₂ slope, which measures ventilation and CO_2_ production, the lower the ventilatory efficiency. Heart rate (HR) and rhythm were continuously monitored throughout the test using a 12-lead electrocardiogram (ECG). Systolic blood pressure (SBP) and diastolic blood pressure (DBP) were measured every 1 min using an automated cuff. Heart rate reserve (HRR), defined as the difference between peak and resting heart rate, is commonly used to assess chronotropic response. To account for the influence of resting HR on maximal HR, an adjusted HRR calculated as (peak HR—resting HR)/(predicted maximal HR—resting HR) is often applied. An adjusted HHR of less than 80% during exercise testing is widely recognized as a diagnostic threshold for chronotropic incompetence (14). Circulatory Power (Peak VO₂ × Peak SBP) assesses cardiac pumping capacity and peripheral vascular resistance. Termination criteria included severe symptoms (angina, dyspnea, dizziness), significant ECG changes (>2-mm ST depression), severe arrhythmias, abnormal BP responses, or the participants' request to stop.

### 2.3 CMR imaging

CMR imaging was performed using a 3.0T MRI scanner (Magnetom Tim Trio, Siemens Medical Solutions) equipped with a 32-channel phased-array cardiac coil. Cine images were acquired in consecutive short-axis slices covering the entire LV, along with standard long-axis views (2-, 3-, and 4-chamber), using a balanced steady-state free precession (bSSFP) sequence during breath-hold. Acquisition parameters included a field of view (FOV) of 320–340 mm^2^, slice thickness of 8 mm with no inter-slice gap, repetition time (TR) of 3.4 ms, echo time (TE) of 1.3 ms, flip angle of 50 °, acquisition matrix of 256 × 144, temporal resolution of 42 ms, and spatial resolution of 1.4 mm × 1.3 mm. Late gadolinium enhancement (LGE) images were acquired approximately 10–15 min after intravenous administration of gadolinium contrast, using a phase-sensitive inversion recovery (PSIR) sequence. The imaging parameters were as follows: FOV, 260 mm × 340 mm; TR, 6.6 ms; TE, 2.0 ms; inversion time (TI), 300–380 ms (adjusted to null normal myocardium); slice thickness, 8 mm; flip angle, 20 °; and matrix size, 116 × 192.

### 2.4 Image analysis

The post-processing software (Medis suit 2.1, QMass 8.1, Medis, Leiden, the Netherlands) was used for image analysis (15). Biventricular end-diastolic volume (EDV), end-systolic volume (ESV), and mass at end-diastole were measured by manually tracing the endocardial and epicardial contours (excluding papillary muscles) on short-axis images. LA diameter was measured at the end of the LV systolic phase on 3-chamber cine images. LA volumes (LAV), including the maximal LA volume (LAV max) at LV end-systole, LA volume at LV diastole before LA contraction (LAV p-ac) and the minimal LAV (LAV min) at LV end-diastole, were acquired by manually tracing LA endocardial borders in 2- and 4-chamber views, excluding the LA appendage and pulmonary veins (16). LGE extent was quantified from the short-axis image stack using a semi-automated approach. Endocardial and epicardial borders were manually traced, and areas of enhancement were identified using a signal intensity threshold set at 6 standard deviations above the mean of remote, normal myocardium. The extent of enhancement was expressed as a percentage of total LV mass. Imaging analyses were performed blinded to CPET results.

### 2.5 Fast LA long-axis strain

LA strain was semi-automatically measured using previously validated fast LA long-axis strain (LA-LAS) analysis, which quantifies strain by tracking the distance between the origins of the mitral valve leaflets and the operator-defined point of the mid-posterior LA wall from standard 2- and 4-chamber cine images (17, 18). The operator manually labelled these points if the automatic tracking algorithm was unable to detect the atrioventricular junction due to slight image artifacts. Using the ventricular cycle, ventricular end-diastole is defined as the zero reference. The corresponding strain curve derived from fast LA-LAS analysis provides three phase-specific parameters: reservoir strain reflects LA expansion during ventricular systole, conduit strain represents passive emptying during early diastole, and booster strain indicates active LA contraction in late diastole. The mean values of LA strain in 2- and 4-chamber views were calculated for further analysis. The intra-rater and inter-rater reproducibility were reported previously (17, 18).

### 2.6 Statistical analysis

Statistical analyses were performed with IBM SPSS Statistics (version 25; IBM Software, IBM Corp), GraphPad Prism (version 8.0; GraphPad Software), and R (version 3.6.0; R Foundation). Categorical variables were summarized as frequencies and percentages, and quantitative variables were expressed as mean ± standard deviation (SD) or median with interquartile range. Kruskal–Wallis test was used to compare quantitative variables among multiple ordered groups. Univariable linear regression analysis was used to assess the association between CPET-derived parameters and other variables. Variables that were statistically significant as well as clinically relevant were tested for collinearity and then sequentially included in multivariable linear regression models to identify independent predictors. Receiver operating characteristic (ROC) curve was used to evaluate the ability of variables in distinguishing various levels of exercise capacity. Bootstrap resampling (1,000 iterations) was performed to assess the stability and internal validity of the regression model estimates. Participants with missing data on key variables (e.g., CPET or CMR parameters) were excluded from the analysis. For all tests, a *P* value <0.05 (2-sided) was considered significant.

## 3 Results

### 3.1 Participant characteristics

A total of 120 patients [60.8% (73/120) male, mean age: 45.5 ± 14.4 years] diagnosed with HCM were included in this study (Table 1). According to the New York Heart Association (NYHA) class, 78.3% (94/120) were in class I/II and 21.7% (26/120) were in class III/IV. Reduced LV function (ejection fraction, EF <50%) was observed in 5.8% (7/120) of patients, while elevated N-terminal pro-B-type natriuretic peptide (NT-pro BNP) levels (>125 pg/mL) were detected in 65.0% (78/120). A total of 89 patients exhibited positive LGE. The mean LVWT and LV mass index were 23.5 ± 6.1 mm and 96.4 ± 39.3 g/m^2^, respectively (Table 2). LVOT obstruction was observed in 71.7% (86/120) of patients, with a median resting LVOT PG of 43.5 mmHg (interquartile range: 20.3–68.3 mmHg) (Table 1).

**Table 1:** Baseline characteristics of the study patients.

Variables	Total HCM (*n* = 120)
Male, *n* (%)	73 (60.8%)
Age, years	45.5 ± 14.4
BMI, kg/m^2^	24.2 ± 3.3
SBP, mmHg	119.7 ± 18.8
DBP, mmHg	74.7 ± 11.3
HR, beats/min	72.6 ± 10.8
Smoking, *n* (%)	21 (17.5%)
Drinking, *n* (%)	21 (17.5%)
Family history of HCM, *n* (%)	20 (16.7%)
HOCM, *n* (%)	86 (71.7%)
Resting LVOT PG, mmHg	43.5 (20.3, 68.3)
LVEF <50%, *n* (%)	7 (5.8%)
LGE, *n* (%)	89 (74.2%)
LGE extent (%)	8.1 (3.9, 13.8)
Comorbidities	
Hypertension, *n* (%)	27 (22.5%)
CAD, *n* (%)	7 (5.8%)
Diabetes, *n* (%)	3 (2.5%)
Medications	
β-blocker, *n* (%)	89 (74.2%)
ACEI/ARBs, *n* (%)	18 (15%)
NYHA functional class	
I/II, *n* (%)	94 (78.3%)
III/IV, *n* (%)	26 (21.7%)
Serum biomarkers	
cTnT, ng/L	16.6 (9.5, 27.3)
High cTnT, *n* (%)	62 (51.3)
NT-pro BNP, pg/mL	886.0 (355.0, 1,944.0)
High NT-pro BNP, *n* (%)	78 (65.0%)
CPET parameters	
Peak VO_2_ (mL/min/kg)	20.6 ± 5.6
Peak VO_2_ < 80% of predict, *n* (%)	91 (77.8%)
AT (mL/min/kg)	11.5 ± 2.7
Peak METs	6.1 ± 1.6
VE/VCO_2_ slope	33.9 ± 8.1
VE/VCO_2_ slope ≥ 34, *n* (%)	37 (30.8%)
HRR (beats/min)	61.7 ± 19.1
Adjusted HRR (%)	63.1 ± 19.5
Chronotropic incompetence, *n* (%)	108 (90.0%)
Circulatory power (mL·mmHg/kg/min)	3,077.0 (2,339.3, 4,048.0)
Circulatory power <4,000, *n* (%)	98 (81.7%)

Data are presented as mean ± SD, *n* (%), or median (IQR). BMI, body mass index; SBP, systolic blood pressure; DBP, diastolic blood pressure; HR, heart rate; HOCM, hypertrophic obstructive cardiomyopathy; LVOT PG, left ventricular outflow tract pressure gradient; LVEF, left ventricular ejection fraction; LGE, late gadolinium enhancement; CAD, coronary artery disease; ACEI, angiotensin-converting enzyme inhibitors; ARBs, angiotensin II receptor blockers; NYHA, New York Heart Association; NT-pro BNP, N-terminal pro-brain natriuretic peptide; cTnT, cardiac troponin T; CPET, cardiopulmonary exercise testing; Peak VO_2_, peak oxygen uptake; AT, anaerobic threshold; METs, metabolic equivalents; VE/VCO_2_, ventilation/carbon dioxide output; HRR, heart rate reserve.

**Table 2:** CMR parameters of study patients stratified by cardiopulmonary function.

Variables	Total HCM (*n* = 120)	Peak VO_2_ (mL/min/kg)				*P* value
		I (≥25, *n* = 21)	II (≥20 ∼ <25, *n* = 47)	III (≥15 ∼ <20, *n* = 37)	IV (<15, *n* = 15)	
HOCM, *n* (%)	86 (71.7)	14 (66.7)	36 (76.6)	26 (70.3)	9 (60.0)	0.488
LGE, *n* (%)	89 (74.2)	14 (66.7)	36 (76.6)	27 (73.0)	12 (80.0)	0.673
LV parameters						
LVMT, mm	23.5 ± 6.1	25.44 ± 5.6	23.9 ± 6.3	22.7 ± 6.1	21.4 ± 5.6	0.202
LVMI, g/m^2^	96.4 ± 39.3	105.33 ± 34.5	92.8 ± 42.2	102.7 ± 39.7	78.9 ± 30.6	0.157
LVEDVI, mL/m^2^	83.6 ± 18.0	83.0 ± 16.4	82.9 ± 19.8	83.3 ± 16.1	88.1 ± 19.9	0.803
LVESVI, mL/m^2^	38.2 ± 22.7	37.0 ± 14.6	36.0 ± 22.8	38.6 ± 23.1	46.6 ± 31.6	0.400
LVEF, %	63.7 ± 9.3	61.7 ± 7.5	66.0 ± 6.1	63.8 ± 10.6	59.0 ± 15.1	0.140
LV long axis strain	−10.0 ± 3.3	−9.4 ± 2.9	−10.7 ± 3.5	−9.3 ± 3.1	−10.6 ± 3.5	0.289
RV parameters						
RVEDVI, mL/m^2^	64.3 ± 14.4	67.7 ± 14.7	63.8 ± 12.4	63.8 ± 16.1	61.9 ± 16.7	0.620
RVESVI, mL/m^2^	25.8 ± 8.9	26.9 ± 9.3	25.7 ± 6.4	24.9 ± 7.9	26.7 ± 16.3	0.691
RVEF, %	62.9 ± 13.6	67.3 ± 14.7	62.1 ± 12.0	62.6 ± 15.6	59.2 ± 11.6	0.579
LA parameters						
LA diameter, mm	42.5 ± 7.4	41.1 ± 7.0	41.53 ± 6.9	42.5 ± 6.30	47.5 ± 10.0	0.170
LAVI max, mL/m^2^	66.9 ± 31.9	60.4 ± 31.0	65.7 ± 29.2	64.1 ± 24.0	88.5 ± 50.2	0.119
LAVI p-ac, mL/m^2^	53.7 ± 24.7	47.4 ± 25.6	54.3 ± 26.8	52.0 ± 20.9	66.4 ± 23.1	0.085
LAVI min, mL/m^2^	40.3 ± 26.2	32.2 ± 23.3	39.2 ± 22.7	39.2 ± 18.7	59.3 ± 46.0	0.033*
LA longitudinal strain, %						
Reservoir strain	21.4 ± 7.3	24.3 ± 6.6	23.0 ± 6.6	19.3 ± 7.9	14.3 ± 3.9	0.001*
Conduit strain	9.5 ± 4.6	10.6 ± 4.4	10.9 ± 4.6	7.7 ± 4.4	7.3 ± 4.0	0.012*
Booster strain	11.8 ± 4.9	13.4 ± 4.6	11.8 ± 4.9	11.3 ± 5.3	9.8 ± 4.0	0.225

Data are presented as mean ± SD or *n* (%). HOCM, obstructive hypertrophic cardiomyopathy; LV, left ventricular; RV, right ventricular; LVMT, left ventricular maximal wall thickness; LVMI, left ventricular mass index; EDVI, end-diastolic volume index; ESVI, end-systolic volume index; EF, ejection fraction; LA, left atrium; LAVI, left atrial volume index; LAVI p-ac, left atrial volume index at pre-atrial contraction.

* *P* < 0.05.

The mean values of Peak VO₂, AT and METs were 20.6 ± 5.6 mL/min/kg, 11.5 ± 2.7 mL/min/kg and 6.1 ± 1.6 METs, respectively (Table 1). CPET abnormalities were common among all included patients with 77.8% (91/120) exhibited reduced exercise tolerance (Peak VO₂ < 80% of predicted), 30.8% (37/120) showed impaired ventilatory efficiency (VE/VCO₂ slope ≥34), 90.0% (108/120) exhibited chronotropic incompetence, and 81.7% (98/120) had impaired cardiac oxygen delivery function (circulatory power <4,000 mL·mmHg/kg/min).

### 3.2 Cardiac function and oxygen consumption

**Figure 1:**
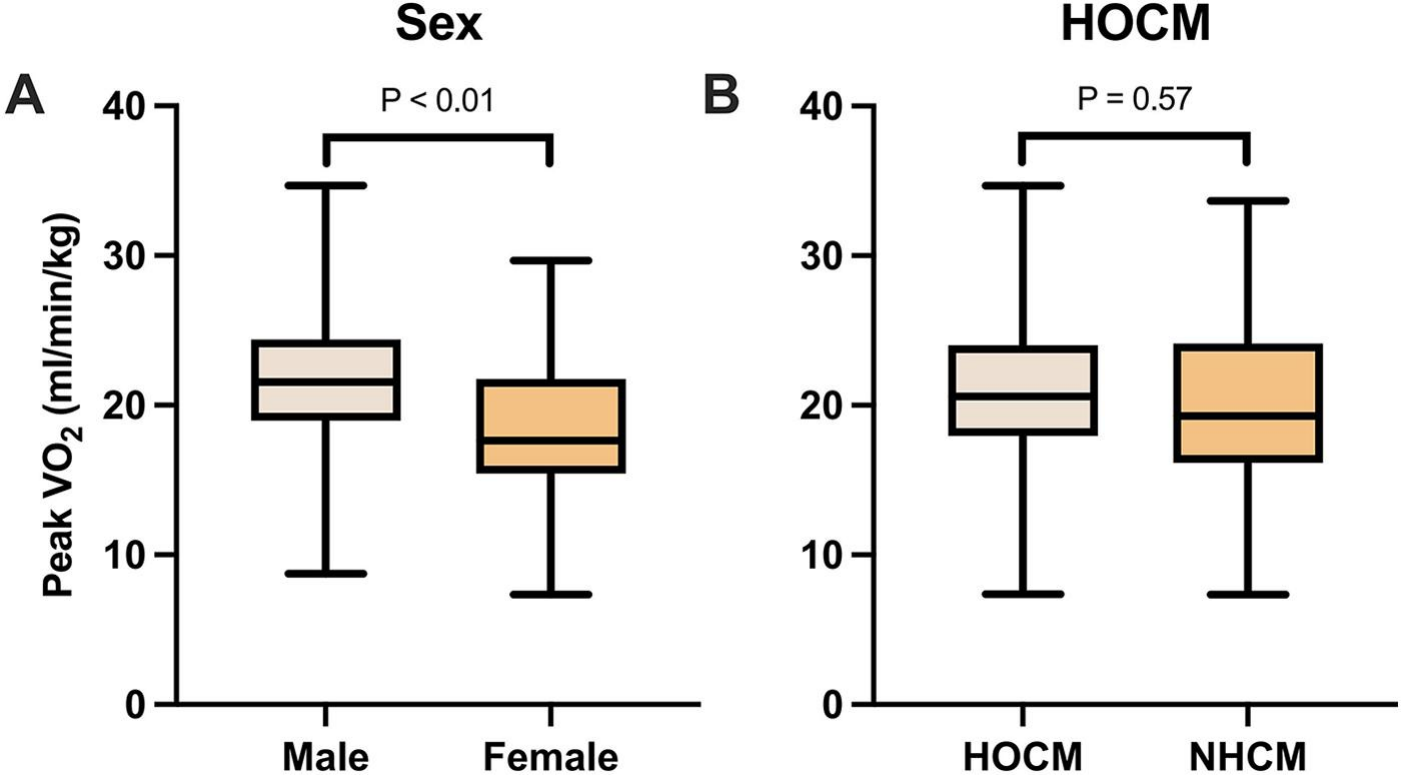
Peak VO₂ between different clinical subgroups. Error bars indicate standard error of the mean. Male patients exhibited higher Peak VO₂ levels (21.9 ± 5.5 mL/min/kg) than females (18.6 ± 5.0 mL/min/kg) **(A)**, while Peak VO₂ did not significantly differ by left ventricular outflow tract (LVOT) obstruction **(B)**. Other abbreviations as in Tables 1, 2.

Peak VO₂ differed significantly by sex. Male patients have higher exercise capacity assessed by Peak VO_2_ (21.9 ± 5.5 vs. 18.6 ± 5.0 mL/min/kg, *P* < 0.01) compared to females (Figure 1A). According to classification standard of the American Medical Association (AMA) (19), patients were stratified into four groups based on Peak VO₂ measured by CPET: Group I (Peak VO₂ ≥ 25 mL/min/kg, *n* = 21), Group II (20 ≤ Peak VO₂ < 25 mL/min/kg, *n* = 47), Group III (15 ≤ Peak VO₂ mL/min/kg < 20, *n* = 37), and Group IV (Peak VO₂ ≤ 15 mL/min/kg, *n* = 15), reflecting a gradient of cardiopulmonary function from good to poor (Table 2). There was a non-significant tendency of LV volumes, right ventricular (RV) volumes, and biventricular EF with worsening cardiopulmonary function (Grade I–IV) (*P* > 0.05). LA diameter showed a non-significant increase with worsening exercise capacity (Grade I–IV: 41.1 ± 7.0 to 47.5 ± 10.0 mm, *P* = 0.170). In contrast, LA volumetric indices showed a more pronounced increase with LAVI max rising from 60.4 ± 31.0 to 88.5 ± 50.2 mL/m^2^ (Grade I–IV, *P* = 0.119), LAVI p-ac rising from 47.4 ± 25.6 to 66.4 ± 23.1 mL/m^2^ (*P* = 0.085), and LAVI min rising significantly from 32.2 ± 23.3 to 59.3 ± 46.0 mL/m^2^ (*P* = 0.033). LA function, assessed by strain analysis, showed a progressive decline, with reservoir strain decreasing from 24.3 ± 6.6% to 14.3 ± 3.9% (*P* = 0.001), conduit strain from 10.6 ± 4.4% to 7.3 ± 4.0% (*P* = 0.012), and booster strain from 13.4 ± 4.6% to 9.8 ± 4.0% (*P* = 0.225).

**Figure 2:**
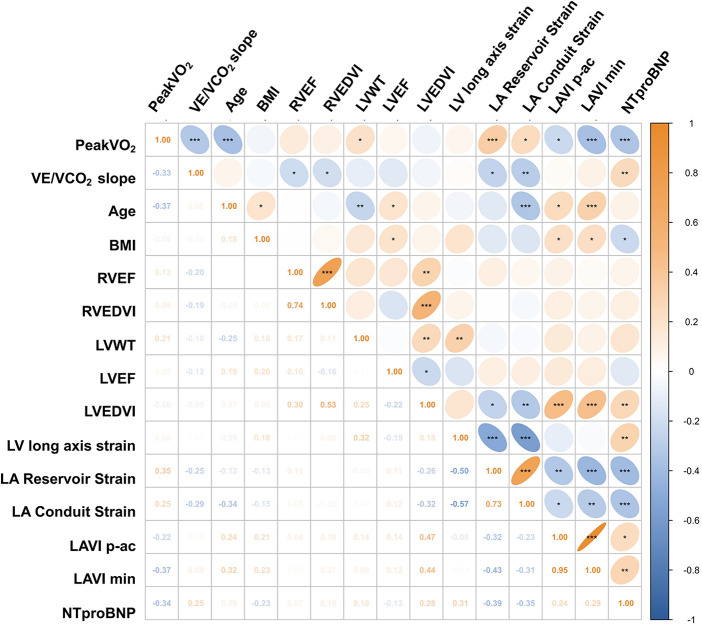
Heatmap of pairwise Pearson correlations between CPET and clinical/CMR variables. *P < 0.05, **P < 0.01, and ***P < 0.001. Abbreviations as in Tables 1, 2.

**Figure 3:**
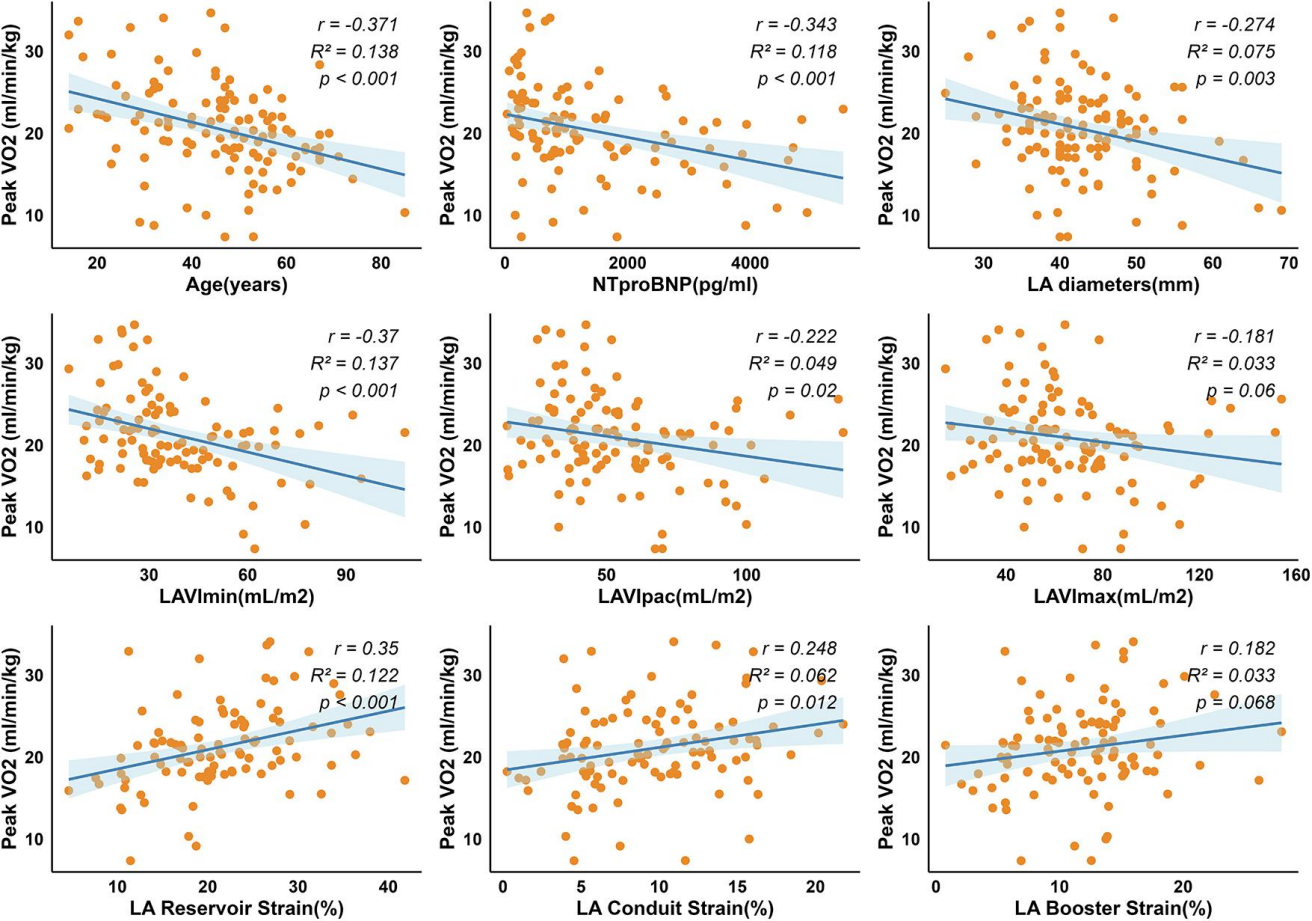
Peak VO₂ correlations with cardiac structural and functional parameters. Dotted lines indicate 95% prediction bands. Peak VO₂ is negatively correlated with age, NT-pro BNP, LA diameters and LAVI, and positively correlated with LA strain. Abbreviations as in Tables 1, 2.

Correlation analyses were performed to evaluate the associations between CPET parameters (including Peak VO₂ and VE/VCO₂ slope), various clinical and CMR parameters (Figures 2, 3). Among clinical factors, age (*r* = −0.37, *R*^2^ = 0.138, *P* < 0.001) and NT-pro BNP levels (*r* = −0.34, *R*^2^ = 0.118, *P* < 0.001) were associated with Peak VO₂. Regarding CMR parameters, LVWT (*r* = 0.21, *P* < 0.05) was the only LV parameter significantly associated with Peak VO_2_. Interestingly, this positive association contrasts with previous studies reporting an inverse relationship. However, after adjustment for age and sex in multivariable linear regression (Supplementary Table S1), LVWT was negatively associated with Peak VO₂, aligning with prior findings. LV related parameters (including volume, LVEF, and strain) showed no significant correlation. In contrast, LA volumes, including LAVI p-ac (*r* = −0.22, *R*^2^ = 0.049, *P* = 0.02) and LAVI min (*r* = −0.37, *R*^2^ = 0.137, *P* < 0.001), were negatively correlated with Peak VO_2_. Additionally, better LA function, reflected by higher reservoir (*r* = 0.35, *R*^2^ = 0.122, *P* < 0.001) and conduit (*r* = 0.248, *R*^2^ = 0.062, *P* = 0.012) were positively associated with Peak VO_2_.

### 3.3 Univariable and multivariable analysis of exercise tolerance

Univariate linear regression analysis identified age, NYHA functional class, NT-pro BNP and LA volume as negative predictors of Peak VO₂, while male sex, adjusted HRR and LA strain were significant positive predictors (all *P* < 0.05) (Table 3). Considering clinical relevance and collinearity, stepwise multiple linear regression analysis included age, sex, NYHA functional class, and adjusted HRR in all models, while LA volume and strain were entered separately. After adjusting for other variables, LA reservoir strain was an independent predictor of Peak VO₂ (*β* = 0.275, *P* < 0.001), superior to LAVI min volumes (*β* = −0.180, *P* = 0.024) (Table 4).

**Table 3:** Univariate analysis of factors associated with peak VO_2_.

Variables	Peak VO_2_	
	*β*	*P* value
Demographic and clinical		
Age	−0.143	<0.001*
Male	3.028	<0.001*
BMI	−0.094	0.544
NYHA functional class		
I/II	−4.912	<0.001*
III/IV		
LGE extend	−0.154	0.097
LGE	0.031	0.744
HOCM	0.049	0.596
Serum biomarkers		
NT-pro BNP	−0.001	<0.001*
CPET Parameters		
Peak HR	0.155	<0.001*
Peak BP	0.017	0.282
Adjusted HRR	0.133	<0.001*
Chronotropic incompetence	−2.650	0.118
LV Parameters		
LVOT PG	0.001	0.943
LVWT	0.184	0.026*
LVMASSI	0.020	0.126
LVEDVI	−0.019	0.515
LVESVI	−0.026	0.250
LVEF	0.031	0.577
LV long axis strain	0.096	0.547
LA Parameters		
LA diameter	−0.206	0.003*
LAVI max	−10.733	0.076
LAVI p-ac	−0.049	0.020*
LAVI min	−0.064	0.001*
LA longitudinal strain		
Reservoir strain	0.236	<0.001*
Conduit strain	0.279	0.012*
Booster strain	0.193	0.068

Abbreviations as in Tables 1, 2.

* *P* < 0.05.

**Table 4:** Multiple linear regression of factors associated with peak VO_2_.

Variables	Multivariable analysis^a^		
	Peak VO_2_		
	*β*	*P* value	Adjusted *R*^2^
Model 1			
Age	−0.296	<0.001	0.395
Male	0.267	<0.001	
NYHA (I–II/III–IV)	−0.206	<0.001	
Adjusted HRR	0.329	<0.001	
LA diameter	−0.149	0.059	
Model 2			
Age	−0.303	<0.001	0.412
Male	0.305	<0.001	
NYHA (I–II/III–IV)	−0.175	0.028	
Adjusted HRR	0.332	<0.001	
LAVI p-ac	−0.152	0.057	
Model 3			
Age	−0.310	<0.001	0.418
Male	0.288	<0.001	
NYHA (I–II/III–IV)	−0.144	0.068	
Adjusted HRR	0.360	<0.001	
LAVI min	−0.180	0.024	
Model 4			
Age	−0.363	<0.001	0.423
Male	0.213	0.008	
NYHA (I–II/III–IV)	−0.184	0.026	
Adjusted HRR	0.285	<0.001	
LA reservoir strain	0.275	<0.001	
Model 5			
Age	−0.283	<0.001	0.386
Male	0.272	<0.001	
NYHA (I–II/III–IV)	−0.203	0.016	
Adjusted HRR	0.301	<0.001	
LA conduit strain	0.156	0.122	
Model 6			
Age	−0.38	<0.001	0.405
Male	0.262	0.001	
NYHA (I–II/III–IV)	−0.213	0.010	
Adjusted HRR	0.306	<0.001	
LA booster strain	0.204	0.011	

Abbreviations as in Tables 1, 2.

a A backward stepwise model with an entry *p* value of 0.05 and a removal *p* value of 0.10, respectively.

**Figure 4:**
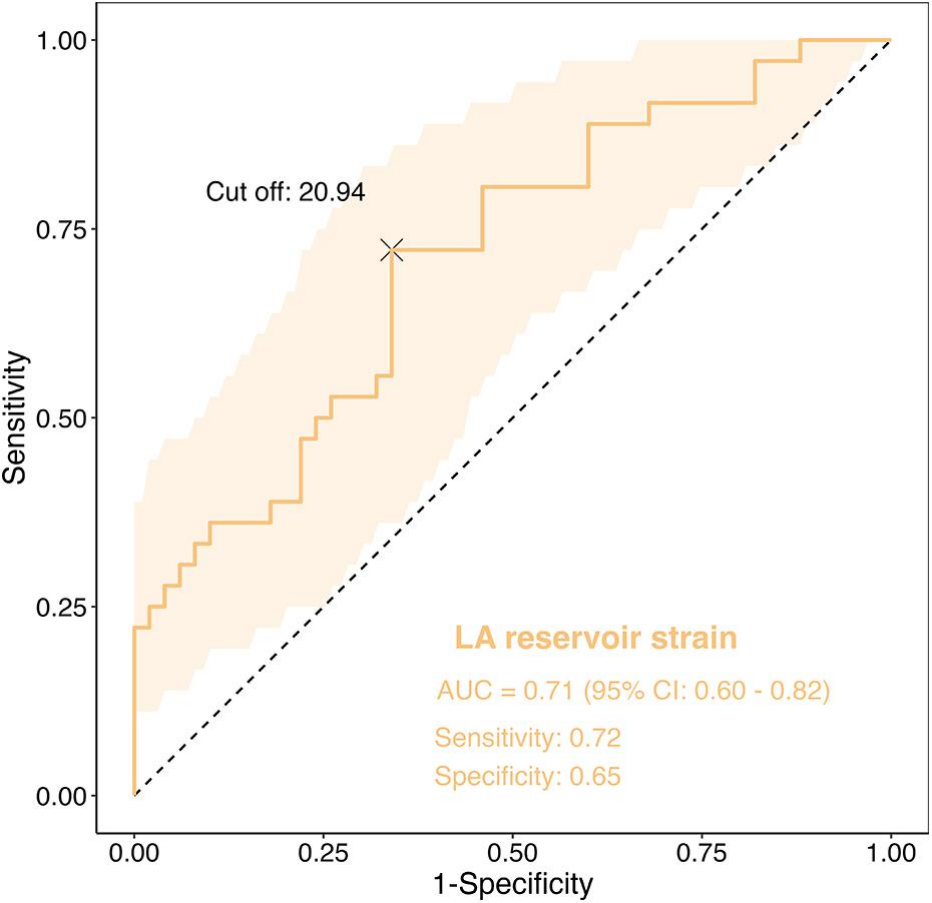
ROC curve for LA reservoir strain in predicting reduced exercise tolerance. LA reservoir strain showed an area under the curve (AUC) of 0.71 (95% CI: 0.60–0.82), with a cutoff of 20.94%, sensitivity of 0.72, and specificity of 0.65.

The ROC analysis further substantiated the meaningful predictive capacity of LA reservoir strain for identifying reduced exercise tolerance (Peak VO₂ < 20 mL/min/kg), with an optimal cutoff value of 20.9%, an AUC of 0.71 (95% confidence interval: 0.60–0.82, *P* < 0.01), a sensitivity of 0.72 and a specificity of 0.65 (Figure 4).

### 3.4 Univariable and multivariable analysis of ventilatory efficiency

Univariate linear regression analysis revealed that RV related parameters (including RVEDVI and RVEF) and LA strain parameters (including reservoir strain and conduit strain) were significantly associated with VE/VCO_2_ slope, while LV related parameters and LA volumes showed no significant association (Supplementary Table S2). Stepwise multiple linear regression analysis identified LA reservoir strain and RVEF as predictors of VE/VCO_2_ slope (Supplementary Table S3).

### 3.5 Performance of different models

An internal validation using 1,000 bootstrap samples was performed to assess the robustness of the multivariable linear regression models predicting Peak VO₂ in Table 4. The adjusted *R*^2^ of the model incorporating LA reservoir strain after bootstrapping was the highest (0.484), indicating its robust and stable performance for predicting exercise intolerance (Supplementary Figure S1).

## 4 Discussion

In this study, we systematically assessed exercise tolerance in patients with HCM using CPET and explored determinants of reduced exercise capacity. The findings can be summarized as follows: (1) LV functional parameters, including volume, EF, strain, and LVOT PG showed no significant correlation with reduced exercise capacity in patients with HCM. (2) LA dysfunction, as reflected by larger LA size and decreased strain, is significantly associated with reduced exercise capacity. (3) LA reservoir strain is an independent predictor of Peak VO_2_, outperforming LA diameter and volumes.

Traditionally, LV systolic dysfunction, especially LVEF, has been considered a major determinant of exercise intolerance. However, in patients with HCM, LV systolic function is generally preserved, and in our study, conventional LV systolic indices showed no significant association with exercise capacity. Similarly, LVOT PG, although widely used to quantify the severity of obstruction in HOCM, was not correlated with Peak VO₂, consistent with previous studies demonstrating its limited role in predicting functional capacity (20). Increasing evidence highlights the role of LV diastolic dysfunction and elevated filling pressure in limiting exercise performance. A retrospective study of 68 HCM patients identified LV diastolic indices, along with age and sex, as independent correlates of Peak VO₂ (21), and Ji-won et al. further confirmed that increased LV filling pressure contributes to impaired exercise capacity independently of myocardial fibrosis or other prognostic markers (5).

Importantly, LA functional impairment may occur even before overt LV diastolic dysfunction, thereby providing earlier insight into the pathophysiological progression. Our analysis confirmed the value of functional assessment of LA in patients with HCM. Furthermore, consistent with previous reports, the LA strain remains significant as a superior indicator than diameter and volume for early functional impairment evaluation (17, 22, 23).

While LA volume reflects the severity of LV diastolic dysfunction, LA dysfunction begins earlier (24). Elevated LV filling pressure imposes an increased afterload on the LA, thereby reducing its reservoir function through mechanical stress on the LA (25). A multi-center study demonstrated that LA reservoir strain has the strongest correlation with LV filling pressure (*r* = −0.52, *P* < 0.001), and LA reservoir strain <18% predicted elevated LV filling pressure better than other conventional parameters, even LA volume (26). This is consistent with our results that LA reservoir strain emerged as an independent predictor of Peak VO₂, demonstrating superior predictive value over LA diameter and volume.

The mechanisms underlying the correlation between LA dysfunction and exercise limitation are multifactorial. In HCM, progressive myocardial hypertrophy, interstitial fibrosis, and LVOT obstruction collectively impair ventricular compliance, leading to elevated LV filling pressure and diastolic dysfunction (27). LA plays a crucial role in cardiac hemodynamics, functioning as a reservoir, conduit, and booster pump to facilitate LV filling and maintain cardiac output. Given its close interaction with LV function, the LA is highly susceptible to chronic volume and pressure overload resulting from increased LV filling pressures (28). The structural and functional alterations of the LA serve as key indicators of LV diastolic dysfunction (27). Previous studies have demonstrated that stroke volume is the major determinant of exercise capacity in HCM. Diastolic dysfunction and increased LV filling pressures reduce the ability to augment cardiac output during exercise (29). Consequently, patients with significant LA dysfunction are unable to generate adequate stroke volume and oxygen delivery, thereby limiting their exercise capacity. In addition to LV, an interaction between RV function and LA mechanics via the pulmonary circulation is physiologically plausible in HCM. Impaired RV systolic function may alter pulmonary hemodynamics, leading to increased pulmonary venous pressure and subsequently increased LA afterload, which may contribute to reduced LA reservoir function (30). However, in our cohort, the resulting LA remodeling and dysfunction, captured by LA strain, may therefore exert a stronger influence on exercise capacity and outweigh the incremental contribution of RV dysfunction when predicting Peak VO₂. In contrast, the impact of RV function may be more directly reflected in ventilatory efficiency parameters such as the VE/VCO₂ slope, and was therefore not included in the Peak VO₂ prediction model.

Effective clinical management of patients with HCM requires both risk stratification for adverse outcomes and assessment of objective disease status. Notably, many HCM patients experience exertional symptoms despite preserved LV systolic function, indicating relying solely on LV systolic parameters or patient-reported symptoms may be insufficient for guiding treatment decisions (5). CPET is recommended for evaluating individualized exercise capacity and exertional heart failure symptoms in HCM patients by measuring Peak VO₂ non-invasively, which is emerged as the strongest independent determinant of exercise tolerance (10, 31). Our study found that several commonly used clinical parameters including LVEF, LVOT obstruction, and patients-reported symptoms were insufficient in discriminating different levels of exercise capacity. In contract, LA function, often overlooked in clinical assessments, shows a stronger association with exercise intolerance than LV parameters. In particular, LA reservoir strain was an independent predictor of Peak VO_2_, highlighting its potential as a surrogate marker for exercise intolerance in HCM patients. These findings suggest that incorporating LA reservoir strain into routine evaluations may enable better clinical management of HCM patients.

## 5 Limitations

Our study has several limitations. First, this is a retrospective analysis in single center with a limited number of included patients. Second, although we identified LA strain as a strong predictor of exercise intolerance, we did not comprehensively assess other potential contributors, such as pulmonary pressures. While resting LVOT gradients were analyzed, exercise-induced LVOT gradients were not evaluated, which may have impacted the interpretation of exercise intolerance. Future studies should comprehensively incorporate more parameters to further clarify the mechanisms of exercise intolerance in HCM. Finally, we did not assess the impact of specific HCM phenotypes on LA function and exercise tolerance.

## 6 Conclusion

In conclusion, LA dysfunction, as reflected by reduced LA strain, was an independent predictors of reduced exercise tolerance in patients with HCM.

## Data Availability

The datasets presented in this article are not readily available due to patient confidentiality requirements. Requests to access the datasets should be directed to the corresponding author, chenyucheng2003@163.com.
