# Supplementary material for: Left atrial function is a strong predictor of exercise intolerance in patients with hypertrophic cardiomyopathy
Source: Front Cardiovasc Med. 2026 Feb 3;13:1717245. doi: 10.3389/fcvm.2026.1717245 (PMC12909542; doi:10.3389/fcvm.2026.1717245)
Supplement: Supplementary file 1 [file Datasheet1.docx]

Supplementary Material

# Supplementary Figures and Tables

## Supplemental Table 1 Multivariable linear regression analysis of the association between LVWT and Peak VO₂

|  | Multivariable analysis* | | |
| --- | --- | --- | --- |
|  | Peak VO_2_ | | |
|  | β | P value | Adjusted R^2^ |
| Age | 0.292 | 0.001 | 0.221 |
| Male | -0.405 | <0.001 |  |
| LVWT | -0.016 | 0.865 |  |

*A backward stepwise model with an entry p value of 0.05 and removal and 0.10, respectively. Abbreviations as in table 1 and 2.

## Supplemental Table 2 Univariate Analysis of factors associated with VE/VCO2 slope

|  | Peak VO_2_ | | |
| --- | --- | --- | --- |
|  | β | P value | |
| **Demographic and clinical** | | | |
| Age | 0.044 | | 0.399 |
| Male | 0.789 | | 0.577 |
| BMI | -0.097 | | 0.672 |
| NYHA functional class |  | |  |
| I/II | 1.406 | | 0.438 |
| III/IV |  |  |  |
| LGE extend | 0.038 | | 0.712 |
| LGE | -0.914 | | 0.620 |
| **Serum biomarkers** |  | |  |
| NT-pro BNP | 0.002 | | 0.008* |
| **CPET Parameters** |  | |  |
| Peak HR | -0.048 | | 0.181 |
| Peak BP | -0.052 | | 0.043* |
| Adjusted HRR | -0.041 | | 0.314 |
| Chronotropic incompetence | 0.022 | | 0.993 |
| **LV Parameters** | | | |
| LVOT PG | -0.021 | | 0.363 |
| LVWT | -0.128 | | 0.309 |
| LVMASSI | 0.003 | | 0.853 |
| LVEDVI | -0.021 | | 0.593 |
| LVESVI | -0.015 | | 0.612 |
| LVEF | -0.093 | | 0.206 |
| LV long axis strain | 0.067 | | 0.771 |
| **RV Parameters** |  | |  |
| RVEDVI | -0.098 | | 0.040* |
| RVESVI | -0.060 | | 0.444 |
| RVEF | -0.107 | | 0.034* |
| **LA Parameters** | | | |
| LA diameter | 0.274 | | 0.006* |
| LAVI max | 0.020 | | 0.378 |
| LAVI p-ac | 0.009 | | 0.755 |
| LAVI min | 0.026 | | 0.328 |
| LA longitudinal strain |  | |  |
| Reservoir strain | -0.257 | | 0.013* |
| Conduit strain | -0.477 | | 0.003* |
| Booster strain | -0.170 | | 0.264 |

* p < 0.05. Abbreviations as in table 1 and 2.

## Supplemental Table 3 Multiple linear regression of factors associated with VE/VCO2 slope

|  | Multivariable analysis* | | |
| --- | --- | --- | --- |
|  | VE/VCO2 slope | | |
|  | β | P value | Adjusted R^2^ |
| LA reservoir strain | -0.33 | 0.002 | 0.163 |
| RVEF | -0.244 | 0.021 |  |

*A backward stepwise model with an entry p value of 0.05 and removal and 0.10, respectively. Abbreviations as in table 1 and 2.

## Supplementary Figure 1. Internal validation of different models


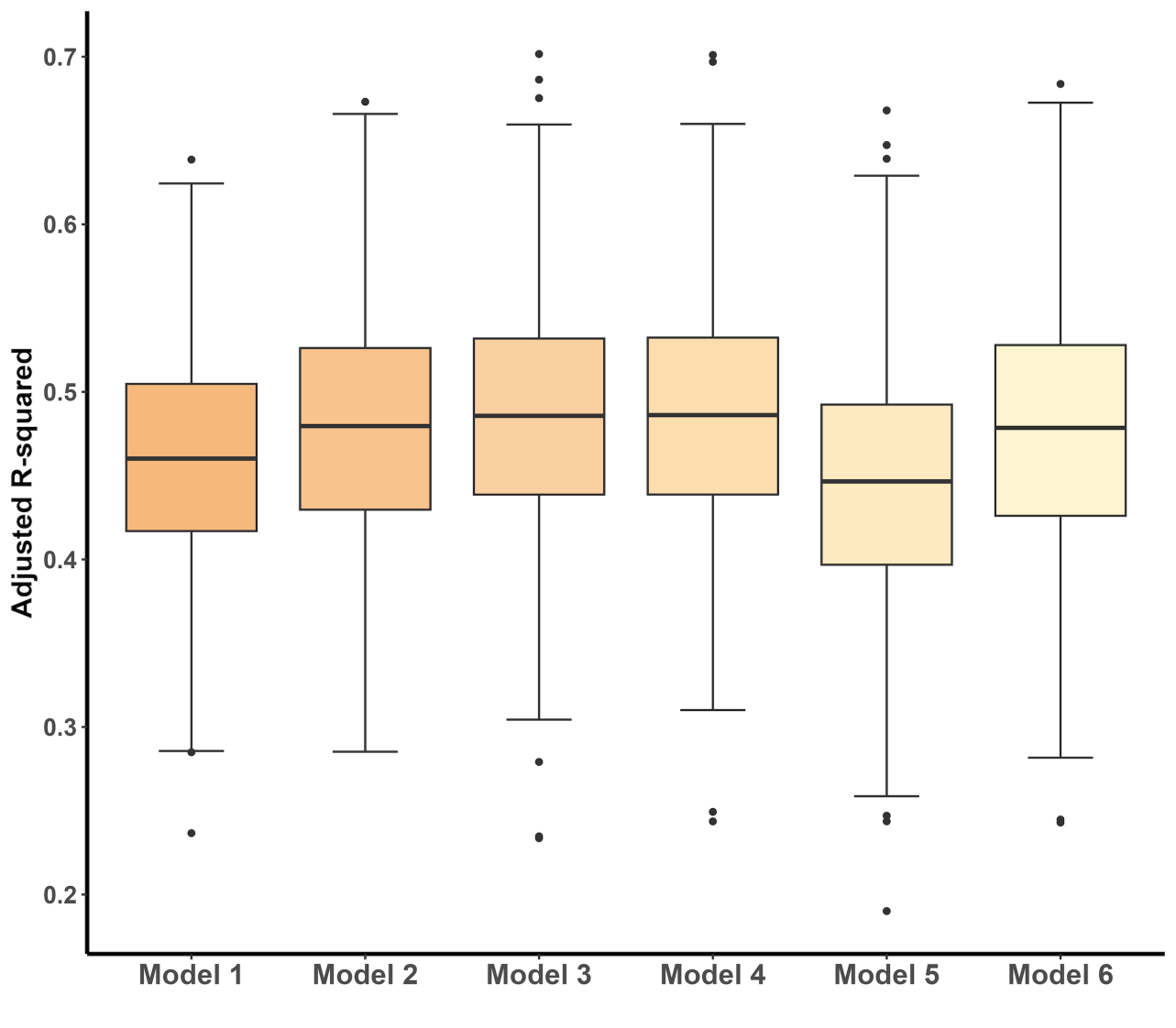
The adjusted R² values after bootstrapping for the different models ranged from about 0.44 to 0.48, with model 4 (including age, sex, NYHA functional class, adjusted HHR and LA reservoir strain) demonstrating the best performance.
